# Development, Progression and Management of Contact Lenses and Eye Care—Editorial Letter

**DOI:** 10.3390/vision7020035

**Published:** 2023-04-22

**Authors:** Nir Erdinest

**Affiliations:** Department of Ophthalmology, Hadassah-Hebrew University Medical Center, Faculty of Medicine, Hebrew University of Jerusalem, Jerusalem 91120, Israel; nir.erdinest@mail.huji.ac.il; Tel.: +972-26777111

The world of contact lenses comprises a considerable segment of the ophthalmology field. Since their invention approximately 135 years ago, the area has evolved unrecognizably when considering technological advances in chemistry, pharmacology, material engineering and imagery. Contact lenses at present hardly resemble the original creation regarding the raw material, internal wetting agents and coatings, the modern cleaning, disinfection and storage methods, and even their target patient [1,2].

Contact lenses can “merely” be a cosmetic alternative to spectacles, a visual acuity enhancer, or a self-esteem rehabilitator affecting social standing and behavior. They can conveniently remove peripheral distortions, increase the visual field, or salvage and restore vision and functionality in patients whose corneas were ravaged by trauma or disease. These advances have provided a leap in the quantity of patient profiles, helped by the modality and exponentially increased quality of care and health preservation [1,2,3].

With all these limitations and contraindications, this fascinating field can positively change the lives of people of all ages, and contact lenses are continuously expanding beyond mere optical accessories. Contact lenses can mechanically or electronically modify their shape to treat presbyopia, manage myopia [4], and detect biological changes such as glucose levels or intra-ocular pressure to monitor diabetes or glaucoma.

More than 18,000 peer-reviewed academic articles have been published on contact lenses. The growth of publications from 1945 to 2022 has been polynomial (Figure 1). Observing the issues since 2010, one can note percentages of 8.85%, 3.75%, 2.16%, 1.79% and 0.7% regarding keratoconus orthokeratology myopia management solutions and therapeutic lenses, respectively (Table 1).

The development of raw materials for lenses is an advanced, dynamic field that uses the most advanced research and technology. Research, clinical trials and the utilization of new materials have directly affected the patient’s quality of life, and they are continuously striving for improvements to lifestyles with ever-increasing visual demands [5]. Artificial intelligence has not overlooked the arena of contact lenses, and more important developments in the field can be expected [6].

Updated contact lens-related developments remains a key consideration for eye care professionals and patients. This Special Issue, “Development, Progression and Management of Contact Lenses and Eye Care”, will focus on therapeutic contact lenses, myopia management, and the common interface between contact lenses and dry-eye disease, inflammation, and contact lens care solutions.

## Figures and Tables

**Figure 1:**
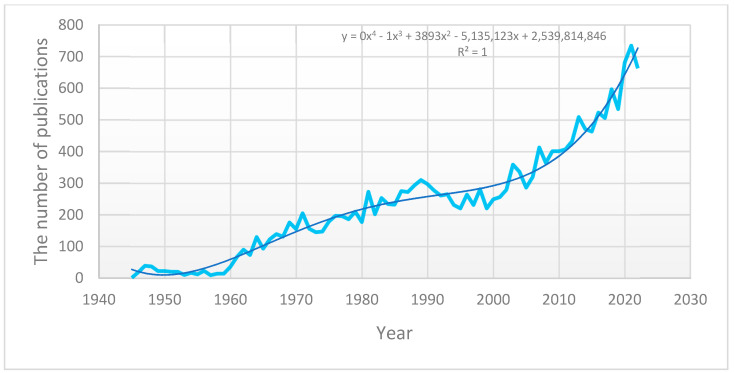
Annual publications of peer-reviewed articles (in PubMed/MEDLINE^®^) between 1945 and 2022. The graph shows a polynomial growth in the 4th order.

**Table 1:** Studies on contact lenses divided by topic conducted from 2010 to 2023, divided into percentages. OK: orthokeratology; CL: contact lens; KC: keratoconus; MGD: meibomian gland dysfunction.

Topics	Percent of Total Articles on Contact Lenses
OK and CL	3.78%
CL and myopia management	2.16%
CL and care solutions	1.79%
CL and therapeutic contact lenses	0.65%
CL and MGD	1.04%
CL and keratoconus	8.85%

## References

[1] Kim J., Cha E., Park J.U. (2020). Recent advances in smart contact lenses. Adv. Mater. Technol..

[2] Willcox M., Keir N., Maseedupally V., Masoudi S., McDermott A., Mobeen R., Purslow C., Santodomingo-Rubido J., Tavazzi S., Zeri F. (2021). BCLA CLEAR-Contact lens wettability, cleaning, disinfection and interactions with tears. Contact Lens Anterior Eye.

[3] Jones L., Hui A., Phan C.-M., Read M.L., Azar D., Buch J., Ciolino J.B., Naroo S.A., Pall B., Romond K. (2021). BCLA CLEAR–Contact lens technologies of the future. Contact Lens Anterior Eye.

[4] Erdinest N., London N., Lavy I., Landau D., Ben Ephraim Noyman D., Levinger N., Morad Y. (2022). Low-Concentration Atropine Monotherapy vs. Combined with MiSight 1 Day Contact Lenses for Myopia Management. Vision.

[5] Erdinest N., London N. (2020). Letter to the Editor Concerning “Historical Development, Applications and Advances in Materials Used in Spectacle Lenses and Contact Lenses”. Clin. Optom..

[6] Ma X., Ahadian S., Liu S., Zhang J., Liu S., Cao T., Lin W., Wu D., de Barros N.R., Zare M.R. (2021). Smart contact lenses for biosensing applications. Adv. Intell. Syst..

